# Polarizer-Free AOTF-Based SWIR Hyperspectral Imaging for Biomedical Applications

**DOI:** 10.3390/s20164439

**Published:** 2020-08-08

**Authors:** Vladislav Batshev, Alexander Machikhin, Grigoriy Martynov, Vitold Pozhar, Sergey Boritko, Milana Sharikova, Vladimir Lomonov, Alexander Vinogradov

**Affiliations:** 1 Scientific and Technological Center of Unique Instrumentation Russian Academy of Sciences, Butlerova Str. 15, Moscow 117342, Russia; machikhin@ntcup.ru (A.M.); martynov.gn@ntcup.ru (G.M.); vitold@ntcup.ru (V.P.); boritko@ntcup.ru (S.B.); sharikova.mo@ntcup.ru (M.S.); 2 Bauman Moscow State Technical University (National Research University), 2-nd Baumanskaya Str. 5, Moscow 105005, Russia; 3 Moscow Power Engineering University (National Research University), Krasnokazarmennaya 14, Moscow 111250, Russia; 4 Moscow Institute of Physics and Technology (National Research University), 9 Institutskiy per., Dolgoprudny 141701, Moscow Region, Russia; 5 Federal Scientific Research Center “Crystallography and Photonics”, Russian Academy of Sciences, Moscow 119333, Russia; yupisarev@yandex.ru (V.L.); aoslab@ntcup.ru (A.V.)

**Keywords:** spectral imaging, hyperspectral imaging, acousto-optical tunable filter, Bragg diffraction, short wave infrared range

## Abstract

Optical biomedical imaging in short wave infrared (SWIR) range within 0.9–1.7 μm is a rapidly developing technique. For this reason, there is an increasing interest in cost-effective and robust hardware for hyperspectral imaging data acquisition in this range. Tunable-filter-based solutions are of particular interest as they provide image processing flexibility and effectiveness in terms of collected data volume. Acousto-optical tunable filters (AOTFs) provide a unique set of features necessary for high-quality SWIR hyperspectral imaging. In this paper, we discuss a polarizer-free configuration of an imaging AOTF that provides a compact and easy-to-integrate design of the whole imager. We have carried out image quality analysis of this system, assembled it and validated its efficiency through multiple experiments. The developed system can be helpful in many hyperspectral applications including biomedical analyses.

## 1. Introduction

Hyperspectral imaging (HSI), also known as imaging spectroscopy, is an increasingly powerful non-invasive method in biomedicine [1,2,3]. It has been proven effective in multiple applications for accurate localization of pathological tissues and quantitative characterization of their spectral properties. Biomedical applications of HSI include intraoperative brain tumor delineation [4], detection of arthritis [5], gastrointestinal tract inspection [6], diagnosis of colon cancer [7], etc. By integrating HSI with microscopy, endoscopy or other optical visualization methods, one can acquire a three-dimensional hyperspectral data cube with proper resolution and extract spatial, spectral, and texture characteristics of the inspected object.

HSI in the short wave infrared (SWIR) range is of particular interest due to lower autofluorescence and scattering of biological tissues. In this range, spectral imaging provides a higher signal-to-noise ratio and higher imaging depth in comparison to those in visible and near infrared ranges. In biomedicine, it is in use for head tissues analysis [8]; studying lymphatic and brain vasculature using fluorescent dye [9]; multiplexed photoluminescence visualization of nanoparticles [10]; some tasks related to lipids, water, or collagen analysis [11]; etc. Acquisition of a hyperspectral data cube may be implemented using different snapshot and scanning techniques [1,12,13,14]. For non-moving objects, spectral scanning by means of an imaging tunable filter seems to be an optimal solution. It provides arbitrary wavelength addressing as well as a good combination of spatial and spectral resolution, acquisition speed and data volume. Acousto-optical tunable filters (AOTFs) based on Bragg diffraction of light by ultrasonic waves seem to be the most promising components for spectral-scanning HSI devices. They provide high spectral (up to 0.1 nm) and spatial (up to 1000 × 1000 resolved elements) resolution, fast (less than 10 µs) and precise wavelength tuning, compactness, no-moving elements design and low power consumption, interactive control of transmission function shape, and programmability. Due to a well-developed technology, AOTFs may be compact and PC-controlled modules, ready for integration into many existing optical schemes. Modern AOTF design methods enable high-throughput and distortion-free imaging, since the methods of proper diffraction geometry choice, accurate AOTF design, and precise optical coupling are well-developed [15,16,17]. As a result, AOTF-based imagers nowadays have numerous applications, in particular for the SWIR range [18,19,20,21,22].

AOTF normally consists of an acousto-optical (AO) crystal located between two crossed polarizers, which are necessary to stop the non-diffracted beam. Polarizing elements increase dimensions, weight, and the cost of the optical system, and hamper the integration of off-the-shelf AOTFs into optical systems. In this regard, a polarizer-free AOTF design, previously demonstrated for non-imaging devices [23], seems especially attractive to be adapted for imaging applications. In this paper, we present an original biomedical SWIR imaging AOTF system, which minimizes aberrations. Such imagers are demanded in microscopy, endoscopy and other biomedical applications. We describe the aberrational analysis of the optical system, the design, and basic technical characteristics. In this article, starting from the physical principles and aberrational analysis, we develop the optimal scheme of the imager and demonstrate its capabilities.

## 2. Theoretical Background

For precise aberrational analysis, the physical principles of the proposed method should be considered. First, we need to determine an appropriate geometry of the Bragg diffraction, then we must define the optimal shape and parameters of the AO cell and, finally, we can estimate the characteristics of the entire optical system.

In non-collinear geometry, the diffracted wave is deflected, so it can be separated from the incident beam without polarizers. In polarizer-free configuration, non-polarized light entering the crystalline AO cell splits into orthogonally polarized ordinary (“o”) and extraordinary (“e”) modes. Both polarization components are selectively diffracted by the same acoustic wave, being deflected in opposite sides with up- and down-conversion of the frequency according to Bragg conditions:

**k_ie_** + **q** − **k_do_** ≡ Δ**k_eo_** ≈ **0**, (1)

**k_io_ − q − k_de_ ≡ Δk_oe_ ≈ 0**.

**Figure 1:**
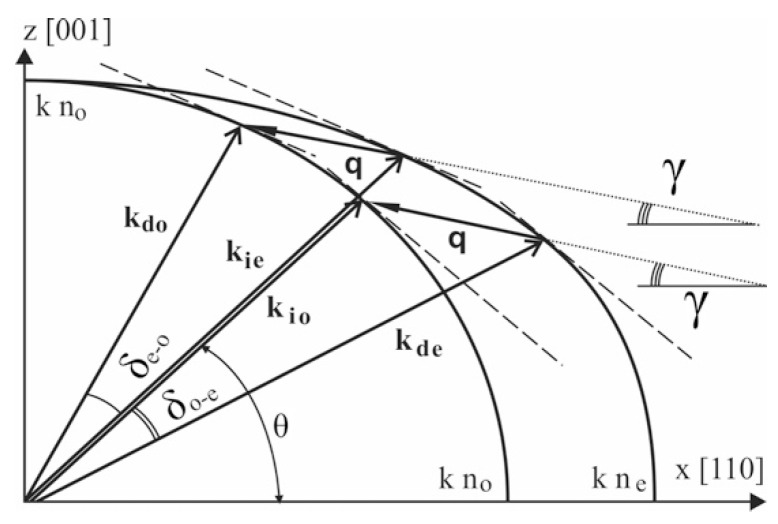
Wave vector diagram of bi-polarization anisotropic acousto-optical (AO) diffraction. **k_io_**, **k_ie_**, **k_do_**, **k_de_**—wave vectors of incident (**i**) and diffracted (**d**) light waves of ordinary (**o**) and extraordinary (**e**) polarization; **q**—wave vector of acoustic wave; **θ**—angle of incidence; γ—angle of acoustic wave propagation, δ_o-e_, δ_e-o_—deflection angles for o→e and e→o modes of diffraction, respectively.

Here, **Δk** is wave-vector mismatch, which should be small enough for effective diffraction, **k_io_**, **k_ie_**, **k_do_**, **k_de_**—wave vectors of incident (**i**) and diffracted (**d**) light waves of ordinary (**o**) and extraordinary (**e**) polarization; **q**—wave vector of acoustic wave. In general case, these conditions are satisfied for optical components of different frequency (or wavelength), but for some angles of incidence θ, which depend on acoustic propagation angle γ, the same wavelength λ is selected for both polarization components (Figure 1). Based on this principle, dual-channel bi-polarization AOTFs can be designed [24,25], which are capable of simultaneous separation of two polarization components (“o” and “e”) of the same wavelength defined by the acoustic wave frequency.

**Figure 2:**
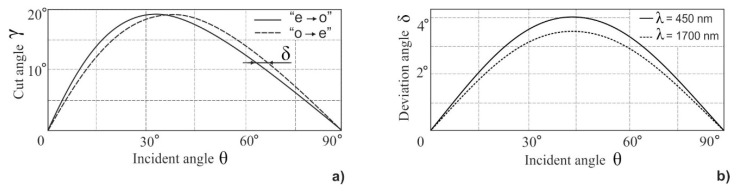
Relationship between angles γ and θ for wide-aperture diffraction in TeO_2_ (**a**) and (**b**) deviation angle dependence δ(θ).

For imaging applications, there is another important requirement: so-called wide-angle or wide-aperture geometry [26]. To achieve a rather large field of view, one needs to provide Bragg condition satisfaction in a wide range of incident angles that are equivalent to parallel tangent geometry. This condition imposes a link upon angles θ and γ for each mode of diffraction: e→o and o→e. As a result, for each incident angle there is an acoustic angle γ, called a crystal cut angle. For each γ, which is fixed for a given AO cell, there are two values of angle θ, which provide non-critical phase matching in some angle range (Figure 2a). The deflection angle (Figure 2b) is almost the same for both modes and depends on incident angle θ and wavelength λ:

(2) $$\delta =\tan^{-1}\left(\frac{n_{e}^{2}}{n_{o}^{2}}\times \tan\theta \right)-\theta .$$

**Figure 3:**
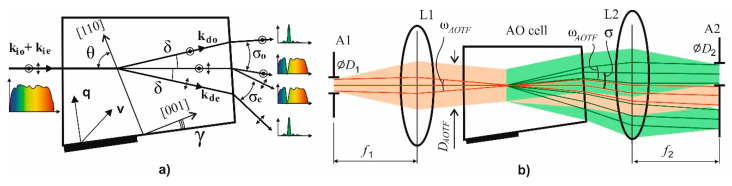
The principle optical schemes of the light beam diffraction and filtration (**a**) and an imaging system based on an acousto-optical tunable filter (AOTF) (**b**). Only one diffracted beam passes through the system, while others are stopped.

As can be seen from Figure 2, the dependences γ(θ) for two modes differ a little. There is a unique value of incident angle θ ≈ 35°, where the plots intersect [27], and with the corresponding cut angle γ ≈ 19.5° the dual-channel bi-polarization imaging AOTF can be designed. Unfortunately, this particular geometry is based on a rather large cut angle γ related to low figure-of-merit *M*_2_ and higher ultrasonic frequencies. That is why in practice, small angles (γ < 10°) are preferred. Thus, we developed a single-channel polarizer-free imaging AOTF, which selects only one linear polarization component, while the other is stopped with a diaphragm as well as a non-diffracted beam (Figure 3).

## 3. Optical System Design and Optimization 

Here, we consider e→o geometry of diffraction, while the other mode o→e can be described in the same way with very similar results.

The principle condition is preventing overlapping of images formed by diffracted and non-diffracted beams. The angular distance between the chief rays of two beams is the out-of-crystal deflection angle σ, so the maximum acceptable angular size of the spectrally selected image ω_AOTF_ should be the same to prevent overlapping:

(3) $$\omega_{AOTF}=\sigma =n\times \delta ,$$

where we omit the subscripts of refraction index *n* and inner deflection angle δ, and also we neglect the small tilt of the back facet of the AO crystal cell [16]. The relation between apertures is determined by the focal length ratio:

(4) $$tan\;\omega_{AOTF}=\frac{D_{1}}{f_{1}}=\frac{D_{2}}{f_{2}}$$

The key characteristic of the system is the number of resolvable image elements, which is limited by the value of the beam divergence ψ. This angle is determined by the size *D_AOTF_* of the beam inside the AOTF formed by entrance aperture (see Figure 3 below):

(5) $$N=\frac{\omega_{AOTF}}{\psi }=\frac{\omega_{AOTF}\cdot D_{AOTF}}{1.22\lambda }=\frac{n\cdot \delta \cdot D_{AOTF}}{1.22\lambda }$$

**Figure 4:**
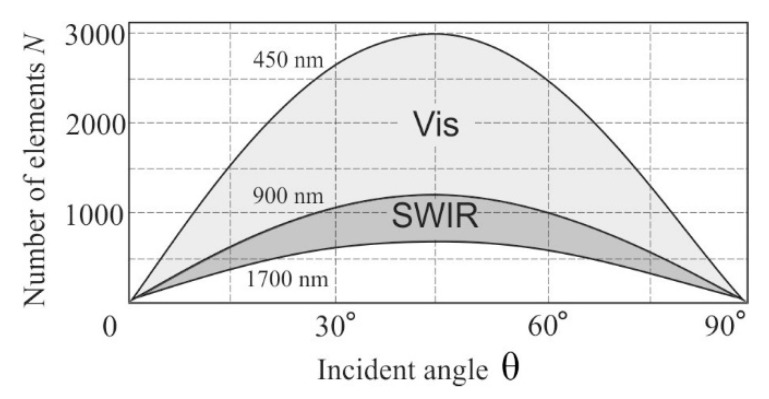
Angle dependence of resolvable element number for visible and short wave infrared (SWIR) ranges for TeO_2_ crystal cell with *D_AOTF_* = 9 mm.

According to Equations (2) and (5), we may conclude that *N* significantly depends on the incident angle θ as well as the wavelength λ (Figure 4). Since the acceptable resolution limit depends on the particular application, the calculated dependence *N*(θ,λ) can be used for a preliminary estimation of the applicability of the polarizer-free imaging AOTF. An aberration analysis of the optical system can be accomplished according to the procedure described in detail in our previous work [16].

## 4. Polarizer-Free AOTF Imager Schemes 

The considered approach to system design is applicable both to collimating and confocal schemes [16]. In the collimating scheme, the input and output diaphragms are field stops, and the rays shown in Figure 3 are the chief rays. In the confocal scheme, these diaphragms are the entrance and the exit pupils, and the rays refer to the axial beam.

**Figure 5:**
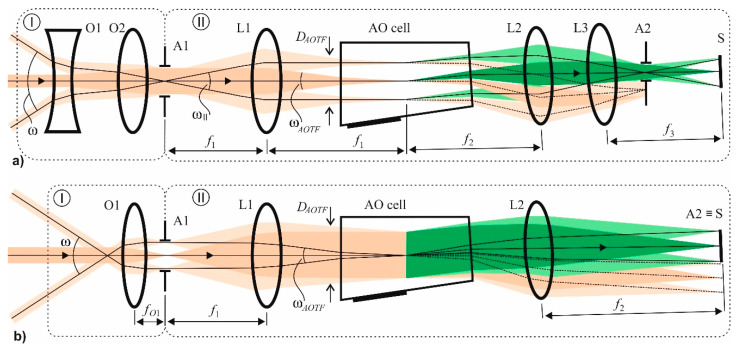
Confocal (**a**) and collimating (**b**) polarizer-free schemes of AOTF-based imagers.

The confocal polarizer-free scheme of AOTF-based hyperspectral imaging system (Figure 5a) consists of an input optical unit I and the primary unit II containing AOTF, optical coupler, and camera. The input unit may vary with respect to imager application. For example, it may be implemented as an afocal system (Figure 5a) consisting of negative O1 and positive O2 lenses and providing magnification necessary for imaging infinite distant objects. Lens L1 focuses the image onto the acoustic grating inside the AOTF. The size of the intermediate image is close to *D*_AOTF_. Lenses L2 and L3 project the image onto sensor S with magnification *m* = *f*_3_/*f*_2_. To cover the sensor area completely, this magnification should be equal to *m* = *S*/*D_AOTF_*. The aperture stop of lens L3 also represents output diaphragm A2.

The input unit I for the polarizer-free collimating scheme (Figure 5b) contains lens O1, which forms an image in the plane of the input diaphragm. The input angular field is defined by the focal length *f*_O1_ of lens O1: $\omega_{AOTF}=2\tan^{-1}\left(\frac{D_{AOTF}}{2f_{O1}}\right)$. Lens L1 collimates light, and L2 focuses it on the sensor. In this scheme, the sensor S serves as the output diaphragm A2. In both schemes, the relationships between *f*_1_, *f*_2_, *D*_1_, and *D*_2_ are described by Equation (4).

Both considered schemes are applied in practice. But the optical aberrations introduced by the AOTF into the image are different in these schemes. For example, the main aberration in a confocal scheme is longitudinal chromatic focal shift. There is also a transverse chromatic image drift, but it can be almost completely compensated for by inclination of the AO cell output face versus the input face. In a collimating scheme, the primary aberration is transverse chromatic drift, and to eliminate it a quite different angle of inclination should be chosen. Thus, each scheme is preferable for particular tasks. Aberration analysis and optimization procedure are described in detail in our previous work [16].

## 5. Results and Discussion

**Figure 6:**
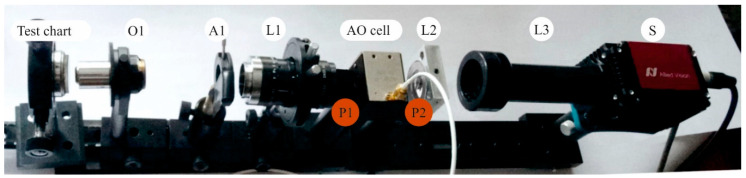
Experimental system. P1, P2—positions for polarizers in reference experimental scheme. (Element designations are presented in text.).

To confirm the theoretical considerations, we assembled the experimental setup shown in Figure 6. The imaging AOTF (γ = 7°, *D*_AOTF_ = 9 mm) for wide-aperture e→o diffraction with angle of incidence θ = 73.9° was designed and manufactured. The spectral range 0.9–1.7 μm was provided by acoustic frequency tuning in the range 30–60 MHz. The spectral bandwidth was 10 nm at λ = 1.06 μm. The deviation angle was δ = 1.8° and the angular aperture of the AOTF was ω_AOTF_ = 4°. The maximum resolution in this case was *N* = 600 at λ = 0.9 μm and *N* = 350 at λ = 1.7 μm. The geometry of the AO crystal cell was optimized for minimization of the image chromatic shift in the confocal scheme shown in Figure 5a. 

For the experiments, we assembled the system shown in Figure 6 with an InGaAs sensor (320 × 256 pixels, 30 × 30 μm^2^ pixel area). To provide optimal image magnification, the microscope objective lens O1 with a focal length 10 mm was used as the input optical unit I. The aperture diaphragm A1 located at the focal plane of the lens L1 provides a telecentric light propagation through the AOTF. Its diameter is *D*_1_ = 5 mm, which ensures the AOTF angular aperture $\omega_{AOTF}=3.8{}^{\circ}$. The focal lengths of the L1, L2 and L3 lenses are 75 mm, 50 mm and 95 mm, respectively. A magnification of the whole system is 14.25 which provides a field of view of 0.54 × 0.67 mm^2^. An intermediate image size inside the AO crystal is 4 × 5 mm^2^.

**Figure 7:**
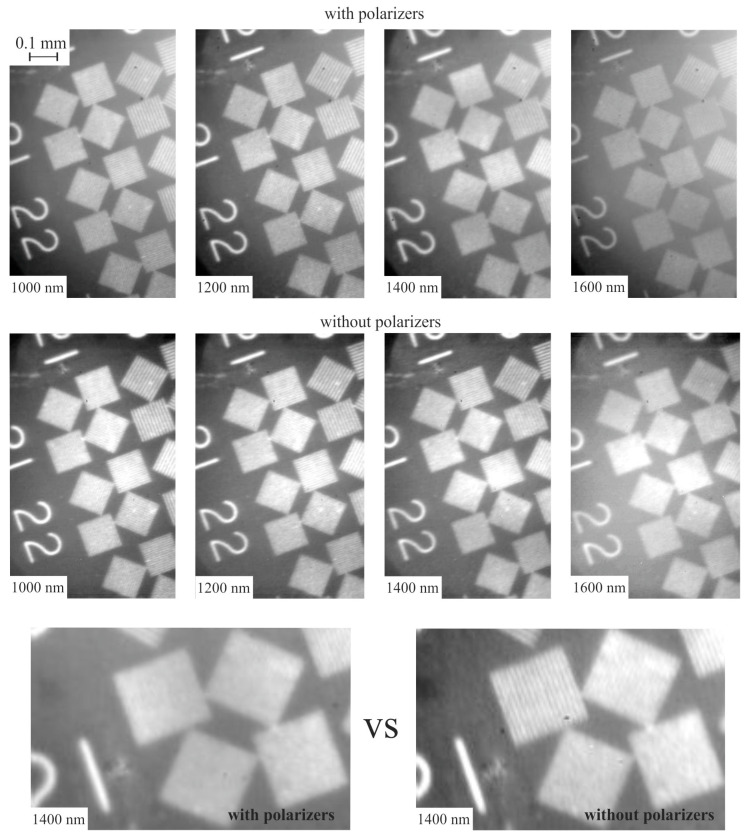
SWIR spectral images of the test chart in the conventional AOTF scheme with polarizers (**top**), and in the polarizer-free scheme (**middle**). Magnified view (**bottom**) for comparison.

To verify the resolution, we recorded the images of the standard test chart (Figure 7). For comparison, we determined the resolution experimentally, both in the polarizer-free configuration ($N_{x}^{0}\times N_{y}^{0}$) and in configuration with the polarizer ($N_{x}^{p}\times N_{y}^{p}$). The theoretical number of resolvable elements *N_x_* × *N_y_* along vertical (*x*) and horizontal (*y*) axes were calculated, taking into account the sensor resolution:

(6) $$\begin{array}{c} N_{x}{}^{-1}=N_{AOx}{}^{-1}+N_{Sx}{}^{-1}\\ N_{y}{}^{-1}=N_{AOy}{}^{-1}+N_{Sy}{}^{-1} \end{array}$$

where *N_AOx_* = *N_AOy_* = *N* is AOTF resolution calculated using Equation (5), *N_Sx_* × *N_Sy_* = 320 × 256 pixels is the sensor resolution. 

Comparison reveals good agreement between experiment and theory (Table 1). The resolution in the polarizer configuration is slightly lower than in the polarizer-free scheme. Moreover, during measurements, it was necessary to increase the exposure time by 1.5 times to obtain the same image intensity as in the polarizer-free scheme.

**Figure 8:**
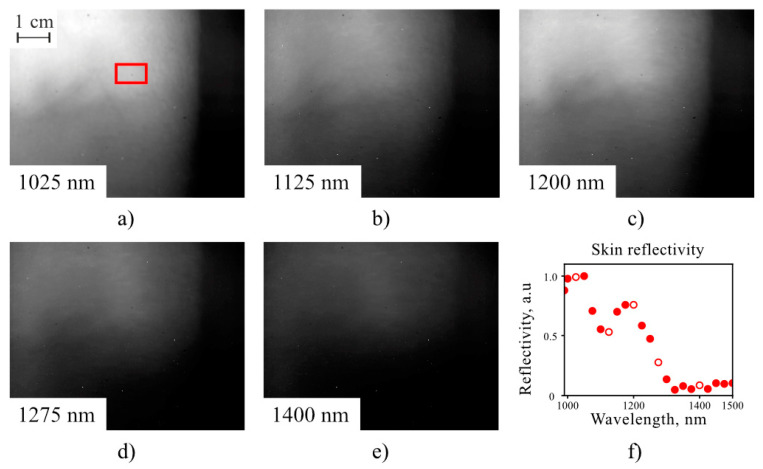
Spectral images (**a**–**e**) of an elbow pit and the reflectivity spectrum (**f**) of the location marked with red rectangle for the region of interest (ROI). The scale bar is 1 cm. Red circles are experimental data. The presented images (**a**–**e**) correspond to the key spectral points depicted with white-filled circles.

To test the system for biomedical applications, we acquired a series of 22 spectral images of a human elbow pit illuminated by an incandescent lamp (Figure 8a–e). The reflectivity spectrum of the region of interest (ROI) was calculated as a ratio of the brightness of each spectral image, averaged over ROI for and normalizing upon the spectral brightness of a white sheet of paper illuminated by the lamp. The spectrum of the human arm measured by the developed system (Figure 8f) exhibits key features of a human skin spectrum demonstrated earlier [28].

## 6. Conclusions

In this paper, we have addressed a single-channel polarizer-free concept of an imaging AOTF. We analyzed the problem of polarizer-free AOTFs and stated the possibility, in principle, of dual-channel imaging AOTF implementation. For a single-channel polarizer-free imaging AOTF, we described the procedure of optical system development and specified its main image quality characteristic, which is the number of resolvable image elements and chromatic aberrations. We created the single-channel polarizer-free imaging system for SWIR biomedical applications, and demonstrated its capabilities experimentally for high-quality spectral image capturing and representation of spectral features.

## Figures and Tables

**Table 1:** AOTF imager resolution.

λ, μm	$N_{x}\times N_{y}$	$N_{x}^{0}\times N_{y}^{0}$	$N_{x}^{p}\times N_{y}^{p}$
1.0	180 × 210	170 × 200	160 × 200
1.2	170 × 200	170 × 200	150 × 200
1.4	160 × 180	150 × 180	140 × 160
1.6	150 × 170	140 × 160	120 × 140
